# Accelerated Tests on Si and SiC Power Transistors with Thermal, Fastand Ultra-Fast Neutrons

**DOI:** 10.3390/s20113021

**Published:** 2020-05-26

**Authors:** Fabio Principato, Saverio Altieri, Leonardo Abbene, Francesco Pintacuda

**Affiliations:** 1 Department of Physics and Chemistry - Emilio Segrè (DiFC), University of Palermo, Viale delle Scienze, Ed. 18, 90128 Palermo, Italy; leonardo.abbene@unipa.it; 2 Department of Physics—University of Pavia and National Institute of Nuclear Physics (INFN), Via Bassi, 6, 27100 Pavia, Italy; saverio.altieri@unipv.it; 3 STMicroelectronics, Stradale Primosole 50, 95121 Catania, Italy; francesco.pintacuda@st.com

**Keywords:** failure in time, power device reliability, silicon carbide, neutron beams, single-event burnout

## Abstract

Neutron test campaigns on silicon (Si) and silicon carbide (SiC) power MOSFETs and IGBTs were conducted at the TRIGA (Training, Research, Isotopes, General Atomics) Mark II (Pavia, Italy) nuclear reactor and ChipIr-ISIS Neutron and Muon Source (Didcot, U.K.) facility. About 2000 power transistors made by STMicroelectronics were tested in all the experiments. Tests with thermal and fast neutrons (up to about 10 MeV) at the TRIGA Mark II reactor showed that single-event burnout (SEB) failures only occurred at voltages close to the rated drain-source voltage. Thermal neutrons did not induce SEB, nor degradation in the electrical parameters of the devices. SEB failures during testing at ChipIr with ultra-fast neutrons (1-800 MeV) were evaluated in terms of failure in time (FIT) versus derating voltage curves according to the JEP151 procedure of the Joint Electron Device Engineering Council (JEDEC). These curves, even if scaled with die size and avalanche voltage, were strongly linked to the technological processes of the devices, although a common trend was observed that highlighted commonalities among the failures of different types of MOSFETs. In both experiments, we observed only SEB failures without single-event gate rupture (SEGR) during the tests. None of the power devices that survived the neutron tests were degraded in their electrical performances. A study of the worst-case bias condition (gate and/or drain) during irradiation was performed.

## 1. Introduction

Since the first observation of single-event burnout (SEB) failure in power MOSFETs exposed to high energy neutrons [1], the hazard to the longevity of these devices due to cosmic radiation, which includes neutrons [2], with energies up to more than 1 GeV [3], has made accelerated neutron testing important.

These tests are important to determine the ultimate device lifetime of power MOSFETs and IGBTs, especially for those with high rated blocking voltages (>300 V), used in several applications such as electrical vehicles, power grids, and avionics [4,5].

The atmospheric neutrons spectrum at sea level covers over twelve decades of neutron energy $E_{n}$, from meV to GeV, with neutron flux of 21 and 13 cm${}^{-2}$h${}^{-1}$ for $E_{n}>1$ MeV and $E_{n}>10$ MeV, respectively. Hence, approximately 40% of the terrestrial neutrons are in the 1-10 MeV energy range [6]. The intensity of cosmic ray-induced neutrons in the atmosphere varies with altitude, increasing from sea level to about 15 km by almost a factor of 1000 and then falling off. Other dependencies, such as on latitude or solar magnetic activity, vary the terrestrial neutron flux, but have less importance for neutron energies greater than 1 MeV. Conversely, at lower energies, the flux depends on how local materials scatter neutrons. The atmospheric thermal (i.e., <0.4 eV) neutron flux at sea level varies with the location and ranges from 6.6 up to 10 cm${}^{-2}$h${}^{-1}$ with an average of 8.2 cm${}^{-2}$h${}^{-1}$ [7].

Thermal neutrons, through the ${}^{10}$B(n,α) reaction, can switch on the parasitic BJT of the MOSFET with subsequent SEB activation [8]; typically, ${}^{10}$B is present in the dopant of the p-body region of the silicon n-channel power MOSFETs and BPSG (borophosphosilicate glass) passivation layer. To the best of our knowledge, few studies are dedicated to the tests of power devices under thermal neutrons [9].

Fast and ultra-fast neutrons can cause different failure mechanisms in power MOSFETs and IGBTs. The most common failure mechanisms are SEB and single-event gate rupture (SEGR) [8,10,11,12,13,14,15]. Neutron lattice collisions produce recoil atoms or spallation products that create electron-hole pairs along its trajectory through the lattice. These charge-plasmas may turn on the parasitic bipolar junction transistor, which leads the device from its normal off-state blocking voltage to its second breakdown state [16] or it settles in the sensitive volume of the device such as the epi/substrate junction [17]. Both mechanisms lead to SEB failure. The contribution of the parasitic bipolar junction in the SEB activation due to atmospheric neutrons seems to be essential for destructive device failure for MOSFETs and IGBTs with a voltage rating lower than 600 V [4].

Power electronic devices that are vulnerable to terrestrial cosmic radiation, such as MOSFETs, IGBTs, and diodes with the minimum nominal blocking voltage of 300 V [18], are subject to accelerated neutron testing to estimate the failure in time (FIT) (1 FIT corresponding to on failure in $10^{9}$ device-hours) parameter under different bias conditions [4,10,11,12,13,17]. Several facilities exist, which provide accelerated testing of devices with the high energy atmospheric-like neutron spectrum, such as those at the Los Alamos Neutron Science Center (LANSCE) in the USA and the ChipIr at the ISIS Neutron and Muon Source, Rutherford Appleton Laboratory, U.K. [19]. Both provide neutron flux levels up to $10^{9}$ times the atmospheric flux at sea level. These facilities provide neutron beams with neutron energies of several hundred MeV, which are sufficient to create a localized and dense plasma of electron-hole pairs within the semiconductor layers.

Due to the probabilistic interaction of neutrons with the semiconductor lattice within the device-sensitive volume, often the data of FIT curves of power devices reported in the literature suffer from high levels of uncertainty, which mainly depends on the small number of tested samples. For a confidence level of 95%, the number of fails has to be 10 to determine the failure rate within a factor of two [4]. Due mainly to economic reasons, the number of achieved fails often does not reach this value. Moreover, the FIT data of power devices subjected to neutron irradiation available in the literature often do not include their estimated interval of a certain confidence level, except in a few cases (e.g., [20,21]). The FIT values obtained experimentally and estimated without the interval of their confidence levels can make it difficult to assess the impact of the device technology processes on the neutron radiation hardness of the power devices.

In this work, we present the results of accelerated neutron tests on Si and SiC power MOSFETs and IGBTs, fabricated by STMicroelectronics with different technologies. Thermal and fast neutrons from the TRIGA (Training, Research, Isotopes, General Atomics) Mark II nuclear reactor (Laboratorio Energia Nucleare Applicata (LENA), Pavia, Italy) were used. The facility allows low energy neutrons with a white energy spectrum and fluxes several orders of magnitude greater than those of atmospheric neutrons. We investigated the effects of low energy neutrons on the electrical parameters of the devices and on the probability of the SEB activation under different bias conditions.

Other accelerated neutron tests were performed at the ChipIr facility with high energy neutrons. In this case, the results were analyzed in terms of FIT versus drain voltage curves. We examined how the scaling of these curves by the device active area, and the drift avalanche rating could highlight the impact of technology on the neutron ruggedness of power devices. To this aim, a large number of tested samples allowed us to obtain a reliable and accurate analysis. The impact of the negative gate voltage condition on the failure rate was also investigated.

## 2. Experimental Section

### 2.1. Devices

Table 1 presents the tested devices. These silicon and silicon carbide power MOSFETs and IGBTs were manufactured by STMicroelectronics and are available in the TO247 package.

### 2.2. The TRIGA Mark II Nuclear Reactor

**Figure 1:**
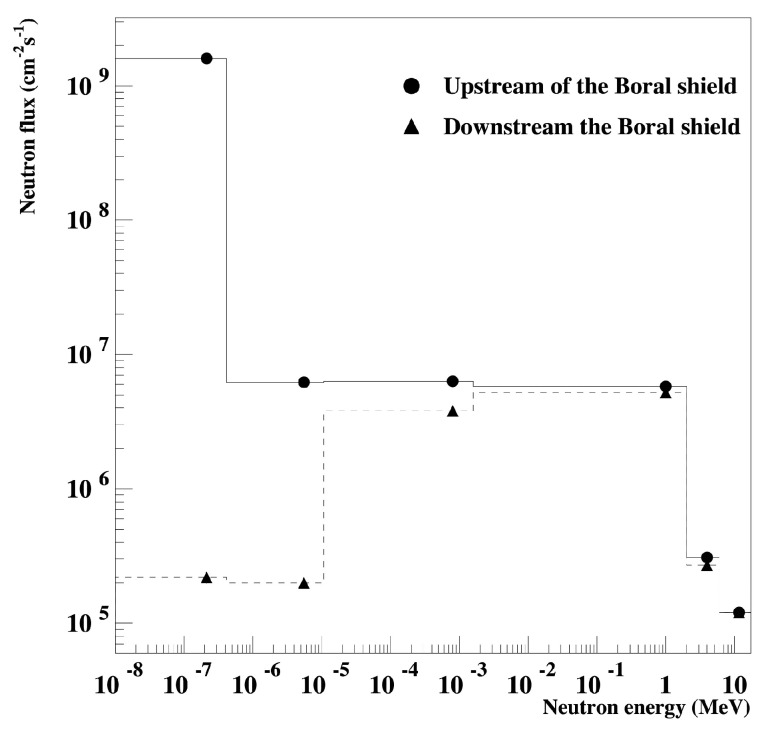
Simulated neutron spectrum upstream and downstream of the Boral shield; the Boral window insertion allows operating with a reduced thermal flux.

The TRIGA Mark II Reactor at LENA of the University (Pavia, Italy) is a water-cooled and water-moderated reactor. At full power (250 kW), the available flux along the core axis is $5\times 10^{13}$ cm${}^{-2}$ s${}^{-1}$. Our boards with the devices under test (DUTs) were located at the end of the thermal column, where the neutron beam comes out of a window 20×40 cm wide. A Boral sheet (aluminum and boron carbide particles between two layers of aluminum cladding) can be placed in front of the window to attenuate the low energy neutrons. In Table 2 are shown the neutron flux components before and after Boral shield. The thermal component of the neutron spectrum, in the case the boral shield is present, is greatly reduced by means the neutron+${}^{10}$B reaction, which absorb low-energy (<0.5 eV) thermal neutrons. Neutron flux distributions at the irradiation position shown in Figure 1, were obtained by Monte Carlo simulations using a Monte Carlo N–Particle (MCNP) [22] input file, where the whole reactor structure was described in [23].

### 2.3. The ChipIr Facility

ChipIr is a beamline dedicated to the irradiation of microelectronics with atmospheric-like neutrons. It was built on the second target station of the ISIS Neutron and Muon Source at the Rutherford Appleton Laboratory, U.K. The neutron beam has a spectrum as similar as possible to the atmospheric one with a neutron flux $\approx 5\times 10^{6}$ cm${}^{-2}$s${}^{-1}$ and energies 1−800 MeV. The tested collimator configuration defines a collimated neutron beam of 70 × 70 mm${}^{2}$. More information on our experiment at ChipIr was reported in [24].

### 2.4. The Neutron Tester

For each neutron exposure (run), the devices were tested at the same bias condition and room temperature. The Neutron Tester system allows controlling up to 24 devices per irradiation run. This system was designed according to the JEP151 procedure [18]. The system supplies the bias drain voltage $V_{DS}$ of the device up to 1200 V and the gate bias voltage $V_{GS}$. The system monitors the source $I_{s}$ and gate $I_{g}$ currents of each device during the irradiation test and performs waveform logging with sampling time ≈500 ms. When the $I_{s}$ current exceeds the threshold value $I_{th}$ (fixed to ≈200μA), the relay disconnects the drain power supply. The gate current does not control the relay connected to the power supply. Therefore, anomalous gate current values can be detected by means of the analysis of the gate current waveform data (for example, in the case of SEGR).

The neutron flux measurement was performed with a high-speed counter synchronized with the signals of the facility. At each device SEB failure, the neutron counts were recorded. Every device fail that was identified automatically by the software was afterward controlled to ensure that no other mechanisms except neutron interaction was the root cause. Eight devices under test (DUTs) ware placed in a single board, with stiffening capacitors between the drain and sources.

At the end of each irradiation run, the software performed the calculation of the FIT parameter and the corresponding 95% confidence interval, according to the procedure in [18]. To get narrow confidence intervals, the duration of each irradiation was extended to have at least 3 or 4 failed devices.

## 3. Results and Discussion

### 3.1. Test with Thermal and Fast Neutrons

The following devices were irradiated at the reactor: the MOSFETs SiC_A, SiC_B, Si_A, and Si_D and the IGBT I_B. The devices were tested at different values of $V_{DS}$ voltages and with $V_{GS}=0$ V. Several irradiation runs were performed, with 24 devices in each run and with the reactor at the maximum power of 250 kW. To investigate the effects of the thermal neutrons, we placed the devices, under the same polarization condition, in the two positions of the thermal column: the first without the Boral shield (pre-Boral position with thermal neutrons) and the second in the presence of this shield (post-Boral position, with the attenuated thermal component). The irradiation time for each run ranged from a few minutes up to five hours. The results of these tests showed that the SiC MOSFETs SiC_A and SiC_B and the Si MOSFETs Si_A did not experience either SEB or SEGR failures up to $V_{DS}$ values equal to the rated $BV_{DSS}$, both in the pre-Boral and post-Boral position. In the IGBT I_B SEB and Si MOSFET Si_D, failures occurred when the $V_{DS}$ voltage reached 92% and 86% of $BV_{DSS}$, respectively. The pre-Boral and post-Boral position did not change the failure rate. Hence, in both cases, the SEB fails were induced only by the high energy neutrons. In Table 3, the FIT at the sea level values and the corresponding 95% confidence intervals for the Si_D and I_B devices are shown, assuming that the epithermal and fast neutrons caused the SEB failures. We note the lower FIT values obtained with fast neutrons if compared with those obtained with high energy neutrons (see Section 3.2.3). The mean values of the SEB fluences of the neutrons with energies greater than 1 MeV were $3.6\times 10^{9}$ cm${}^{-2}$ and $1.5\times 10^{10}$ cm${}^{-2}$ for the Si_D and I_B devices, respectively. For lower values of the drain bias, neither device showed fails. For example, the device Si_D at $V_{DS}=350$ V did not fail with a neutron fluence up to $9.8\times 10^{10}$ cm${}^{-2}$. In all cases, for the same $V_{DS}$, the negative $V_{GS}$ voltages bias condition did not change the test results.

The failure detected by the neutron system resulted in the destruction of the power devices. All failed devices had both the gate-source and drain-source shorted. The occurrence of the SEB in the IGBT I_B was not due to the thermal neutrons, because we found the same FIT values in the two positions of the thermal column (upstream and downstream of the Boral shield), where the thermal flux showed a variation of about four orders of magnitude, while the fast component was almost the same.

**Figure 2:**
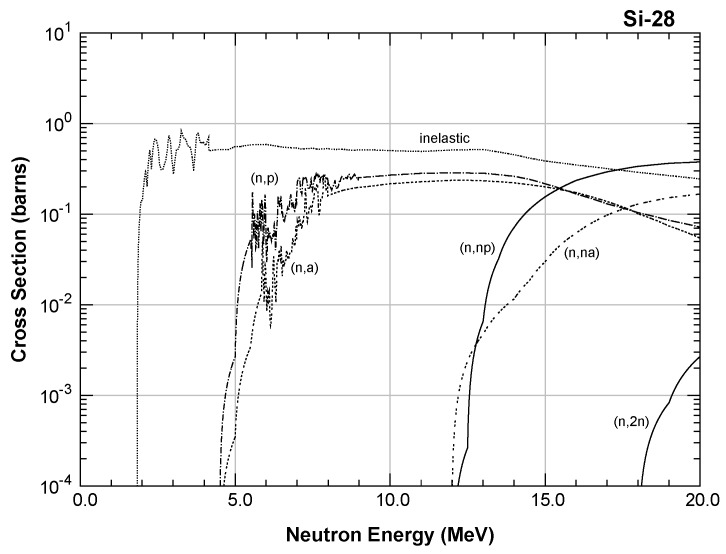
Inelastic scattering and threshold reaction cross-sections at 300 K of isotope ${}^{28}$Si from the Japanese Evaluated Nuclear Data Library [25].

In the energy range of the neutron beam of the reactor, the main nuclear interaction mechanism with silicon nuclei was elastic scattering (but the energy transferred to Si was about 0.07 times the neutron energy). Above ≈1 MeV, a contribution from inelastic scattering started (with gamma emission), and above a few MeV threshold, reactions with high linear energy transfer (LET) charged particles’ emission $\left(n,p\right)$ and $\left(n,\alpha \right)$ occurred; in the thermal energy range, only the radiative n,γ reaction with a typical 1/v cross-section was present, where *v* is the neutron velocity. Figure 2 shows an example of the high energy part of the cross-section for the most abundant isotope ${}^{28}$Si (92.2%). The isotopes ${}^{29}$Si (4.7%) and ${}^{30}$Si (3.1%) showed a similar behavior.

Probably, the $\left(n,\alpha \right)$ and $\left(n,p\right)$ threshold reactions in our test were responsible for the SEB failures in both the Si_D MOSFET and I_B IGBT. By supposing that the neutrons with energies in the 1–10 MeV range caused the SEB in these devices, we calculated the FIT at sea level of these devices, by assuming that in this energy range, the neutron flux at sea level was 8 cm${}^{-2}$ h${}^{-1}$. The results are shown in Table 3.

**Figure 3:**
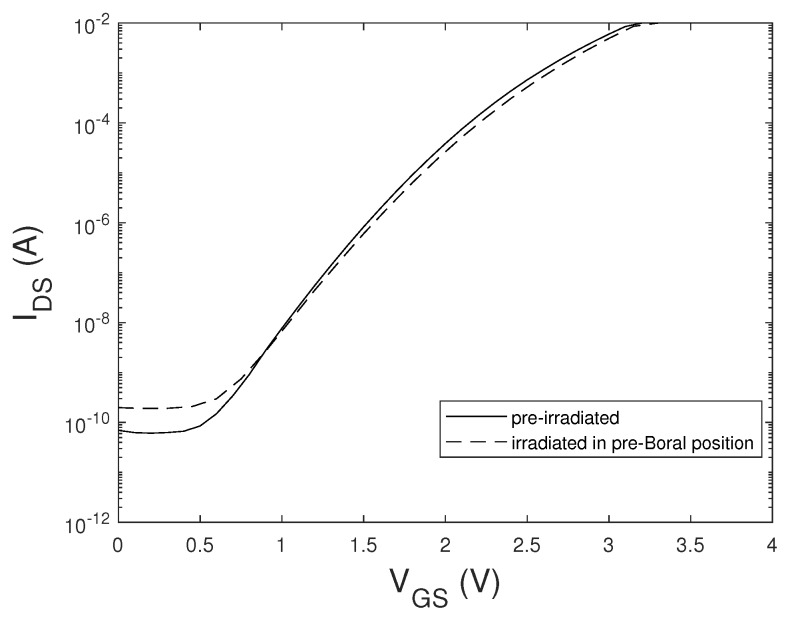
$I_{DS}-V_{GS}$ curves at $V_{DS}=2.0$ V of a SiC_B MOSFET sample before and immediately after irradiation with thermal and fast neutrons (pre-Boral position).

The devices that survived the tests, even those exposed to the thermal component, did not show any significant degradation in their electrical parameters. In Figure 3, the curves of the $I_{DS}-V_{GS}$ curves of a SiC_B power MOSFET sample that did not fail during the neutron irradiation test in the pre-Boral position are shown, where the thermal component of the neutron was not attenuated. We only note a slight increase in the leakage $I_{DS}$ current. This increase of the leakage current could not be due to the gamma dose absorbed by the device, which was in the worst case of the order of a few Gy(Si), thus not enough to cause total dose effects on the devices [26]. The estimated value of the gamma dose absorbed by the devices was performed based on the value of the gamma dose rate generated by the interaction of the thermal neutrons with the Boral shield, which was 1.6 ± 0.1 Gy/h with a 250 kW power reactor.

**Figure 4:**
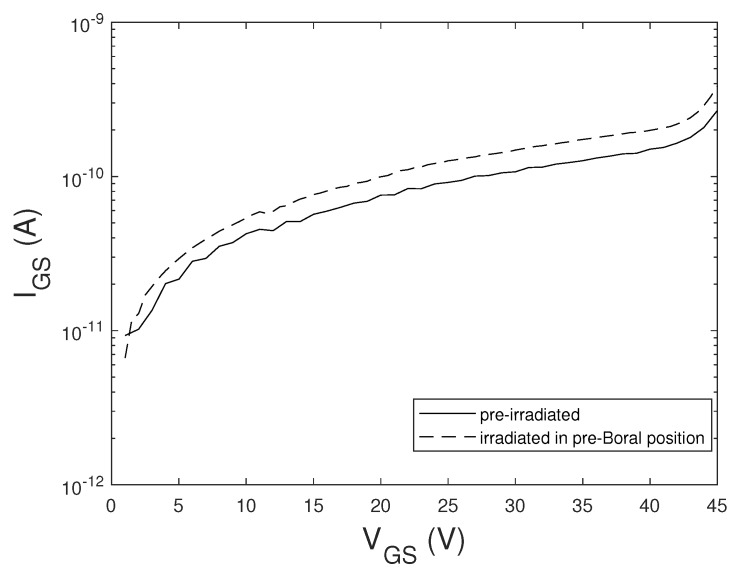
$I_{GS}-V_{GS}$ curves at $V_{DS}=0$ V of a I_B IGBT sample before and immediately after irradiation with thermal and fast neutrons (pre-Boral position) with $V_{GS}=+40$ V.

To test the effects of the thermal neutrons on the integrity of the gate oxide, some samples of MOSFET and IGBT were irradiated at $V_{GS}=+40$ V and $V_{DS}=0$ V. In this bias condition, the gate voltage is over its maximum rated value and close to the Fowler-Nordheim onset. This high electric field present in the oxide promotes charge trapping, due to the interaction of γ-rays and neutrons with the oxide. Figure 4 shows the curves of the gate leakage current of IGBT sample before and immediately after irradiation with fluence $2.9\times 10^{13}$ cm${}^{-2}$ of thermal neutrons. After this exposure the devices were radioactive with a dose-rate at contact in the order of tens mSv/h. Figure 4 shows that the gate current does not have significant variation due to the interaction with neutrons and gamma-ray up to the Fowler-Nordheim knee.

### 3.2. Neutron Test at the ChipIr Facility

At the ChipIr Facility all power devices in Table 1 have been irradiated. Before irradiation, each device was subject to multiple electric tests, namely determining the breakdown voltage, the gate and drain leakage current, and the sub-threshold curves. Several irradiation runs were performed, with a total of three test boards (24 devices) in each run, where the irradiated devices have the same bias condition. The irradiation tests were performed by placing these boards one after the other in the neutron beam. The attenuation in neutron beam flux due to boards is negligible.

#### 3.2.1. Analysis in the Time Domain

The neutron test at the ChipIr facility with ultra-fast neutrons of the SiC and Si MOSFETs and IGBTs resulted in the destruction of the power devices that failed during the test, with both being drain-source and gate-source shorted. The analysis of the gate and drain current did not reveal any increase in either current before the SEB events. The sampling time of our Neutron Tester (≈500 ms) was not able to follow the transient of the currents during the failure, usually characterized by a time duration of less than 10 ns [13].

#### 3.2.2. Analysis of Degradation Phenomena

In the investigated power devices irradiated at different drain bias values, with $V_{GS}$ at zero or negative voltage, no SEGR failures were observed during all the runs. Only SEB failures occurred. Power devices that survived the tests did not show degradation in the electrical parameters. This was verified by electrical characterizations (sub-threshold and gate leakage curves not shown) performed before and after irradiation. Moreover, we performed irradiation runs with eight SiC_A devices at the bias conditions $V_{DS}=0$ V and $V_{GS}=+25$ V. This extreme bias condition was used in our tests only to investigate possible degradation phenomena in the gate oxide due to the ultra-fast neutrons. At this bias condition, the electric field in the gate oxide was ≈5 MV/cm. After neutron fluence up to $\approx 5\times 10^{11}$ 1/(cm${}^{2}\cdot$device), neither SEGR during irradiation nor degradation after the test were observed in the gate oxide.

#### 3.2.3. Failure Rate Analysis

**Figure 5:**
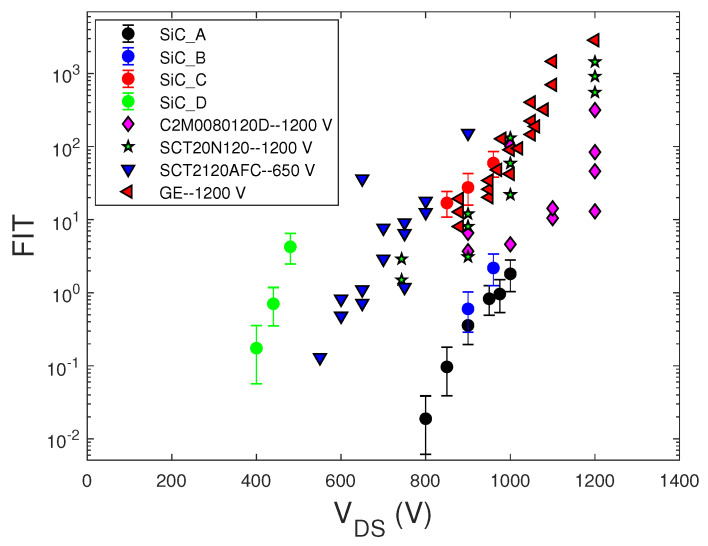
FIT data at sea level of the STMicroelectronics SiC MOSFETs (•) with the 95% confidence intervals. Data of the commercial SiC MOSFETs C2M0080120D, SCT20N120-1200 V, and SCT2120AFC-650 V from [13] and GE-1200 V from [27].

Figure 5 shows the FIT results at sea level versus the bias drain voltage (at $V_{GS}=0$ V) of SiC MOSFETs with their 95% confidence intervals. For comparison, in the same figure, the data of the commercial SiC MOSFETs C2M0080120D-rating 1200 V (Cree), SCT20N120-1200 V (STMicroelectronics), SCT2120AFC-650 V (ROHM) reported in [13], and GE-1200 V (GE) from [27] are shown.

**Figure 6:**
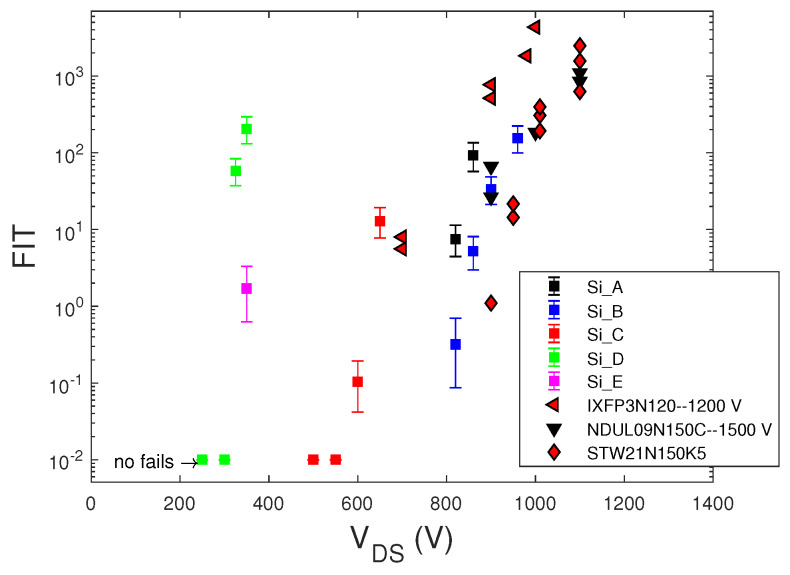
FIT data at sea level of the STMicroelectronics Si MOSFETs (□). Data of the commercial Si MOSFETs IXFP3N120–1200 V (Ixys), NDUL09N150C -1200 V (ONSEMI), and STW21N150K5-1500 V (STMicroelectronics) from [13].

Figure 6 shows the measured bias-dependent FIT data at sea level of STMicroelectronics Si MOSFETs with the data of some commercial Si MOSFETs from [13].

**Figure 7:**
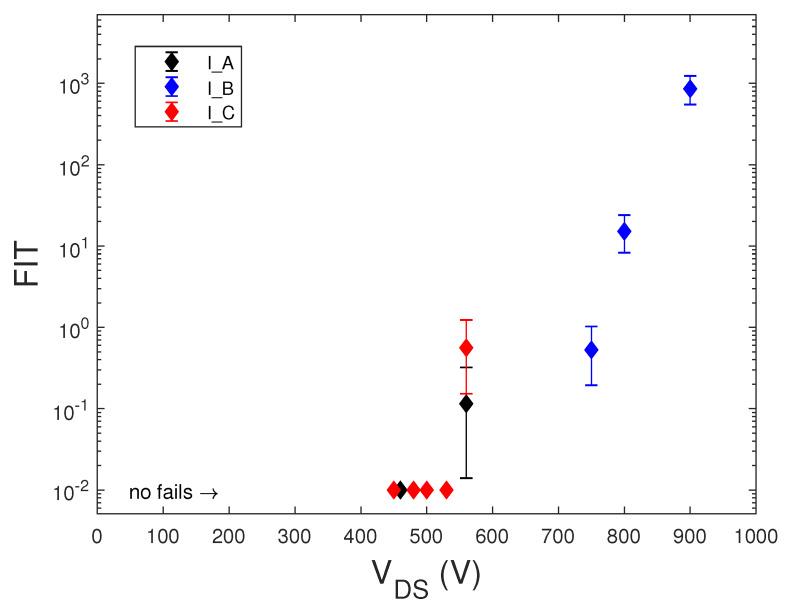
Measured FIT data at sea level of the STMicroelectronics IGBTs.

Figure 7 shows the measured bias-dependent FIT data at sea level of the STMicroelectronics IGBTs.

The FIT data of commercial MOSFETs from [13,27] shown in both Figure 5 and Figure 6 did not include the confidence intervals. This lack made the comparison between different devices inaccurate. In some cases, for the same $V_{DS}$ bias value, the FIT values obtained in different irradiation runs are reported, rather than showing their average value at the same bias.

We note that SEB failure probability increased exponentially with the applied voltage for both SiC and Si MOSFET and IGBTs devices, but only silicon devices showed a threshold drain voltage, below which no fails occurred. Furthermore, over this threshold voltage, the FIT of Si MOSFETs increased faster with the voltage drain than that of SiC MOSFETs. The lack of this threshold voltage in SiC MOSFETs could be due to defects in the SiC material, which anticipated the achievement of the peak electric field needed to sustain avalanche multiplication with the regenerative feedback condition leading to SEB.

Comparing SiC and Si power MOSFETs with the same voltage rating, we note that SiC devices exhibited significantly lower failure rates than those of Si MOSFETs, thus confirming this general characteristic previously observed in [12,21] and explained by considering the smaller sensitive volume and the lower gain of the parasitic bipolar junction of the SiC devices with respect to that of the Si ones.

**Figure 8:**
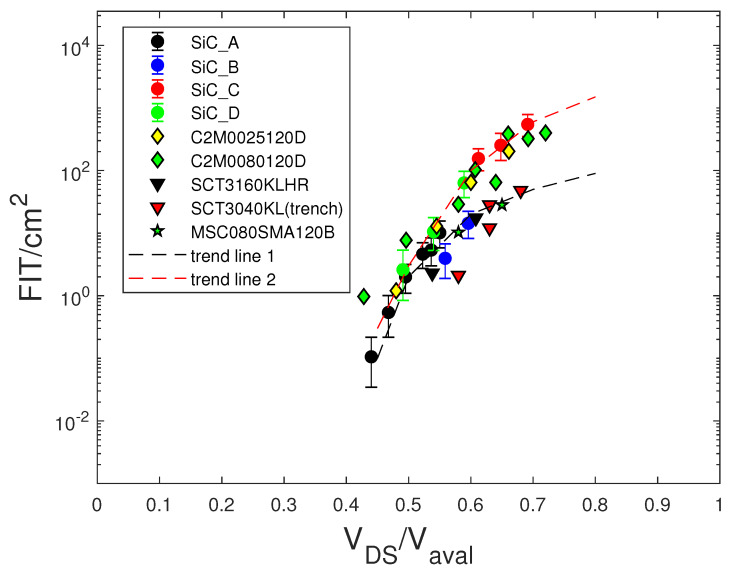
Measured (•) FIT data at sea level of SiC MOSFETs normalized to the active area versus $V_{DS}/V_{aval}$. Data of the commercial SiC MOSFETs C2M0025120D (Cree), SCT3160KLHR -1200 V (ROHM), SCT3040KL-Trench (ROHM), and MSC080SMA120B (Microsemi) from [11].

In [11,14,15], the authors asserted that the FIT values (per device active area, FIT/cm${}^{2}$) of SiC power MOSFETs versus the $V_{DS}$ voltage scaled to the avalanche voltage ($V_{aval}$) of the device obeyed a universal trend. To verify the validity of this behavior, we show in Figure 8 the FIT/cm${}^{2}$ versus $V_{DS}/V_{aval}$ curves for the tested devices and some devices reported in [11]. We note that at least two trend line curves existed and that the device C2M0080120D overlapped both curves. In particular, we note that at $V_{DS}/V_{aval}\approx 0.6$, the confidence intervals of the devices SiC_B and SiC_D did not overlap.

Moreover, at higher $V_{DS}/V_{aval}$ values, the differences between some devices became more remarkable. Hence, the data shown in Figure 8 did not prove the existence of a universal trend attainable for the SiC material for the investigated devices. Therefore, we concluded that the FIT/cm${}^{2}$ versus $V_{DS}/V_{aval}$ curves could highlight differences in the neutron radiation hardness of MOSFETs, which depended on the technology and the design of the device. Indeed, the FIT/cm${}^{2}$ values of the SiC_C MOSFETs were significantly higher than those of the SiC_A and SiC_B SiC MOSFETs. These improvements obtained in SiC_A and SiC_B devices were due to the change of some technological processes of the SiC_C device, which led both devices to have better electrical parameters and greater hardness against cosmic rays.

The FIT/cm${}^{2}$ versus $V_{DS}/V_{aval}$ data (not shown) of the investigated Si MOSFETs and IGBTs did not follow a common trend, although general models for cosmic ray-induced failures in silicon power devices exist [28].

**Figure 9:**
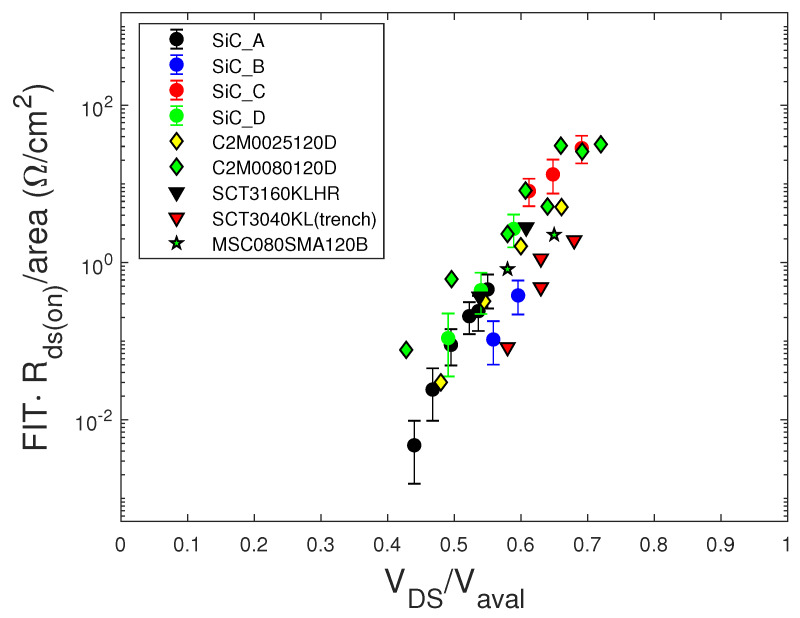
FIT$\cdot R_{ds(on)}$/cm${}^{2}$ data versus $V_{DS}/V_{aval}$ of the samples of Figure 8.

To better compare SiC MOSFETs with different technologies, we scaled the FIT/cm${}^{2}$ values of Figure 8 by the $R_{ds(on)}$ of the devices. In Figure 9, the FIT$\cdot R_{ds(on)}$/cm${}^{2}$ versus $V_{DS}/V_{aval}$ curves are shown. Although the differences between the various devices seemed better mitigated with respect to those of Figure 8, the dependence on the technology of the devices persisted.

**Figure 10:**
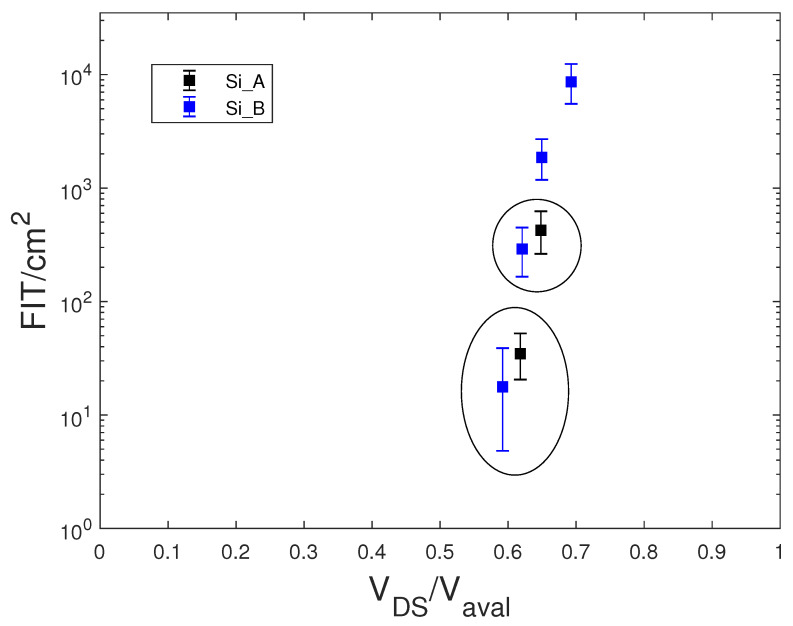
Measured FIT/cm${}^{2}$ versus $V_{DS}/V_{aval}$ of equal Si MOSFETs with different chip sizes.

Figure 10 shows FIT/cm${}^{2}$ versus $V_{DS}/V_{aval}$ curves for the Si_A and Si_B MOSFETs. It is well known that the FIT value is proportional to the active area of the devices. The Si MOSFETs Si_A and Si_B were devices made with the same technology, but with different chip sizes. We observed that the ratio of the FIT values, shown in Figure 6, measured at the same $V_{DS}$ was roughly equal to the ratio of the chip size area. Conversely, the circled points in Figure 10 correspond to the same value of the $V_{DS}$ voltage, 820 V and 860 V, respectively, but the FIT/cm${}^{2}$ values of these devices for the same $V_{DS}/V_{aval}$ value did not overlap as expected. This occurred because the $V_{aval}$ values of the Si_A samples were slightly lower (a few tens of volts) than those of the Si_B devices. Therefore, the curves FIT/cm${}^{2}$ versus $V_{DS}/V_{aval}$ showed differences that did not depend on the technology, but on factors that were linked to the typical spread of the technological processes. Hence, these curves could blur the impact of the technology processes over the hardness against cosmic rays.

**Figure 11:**
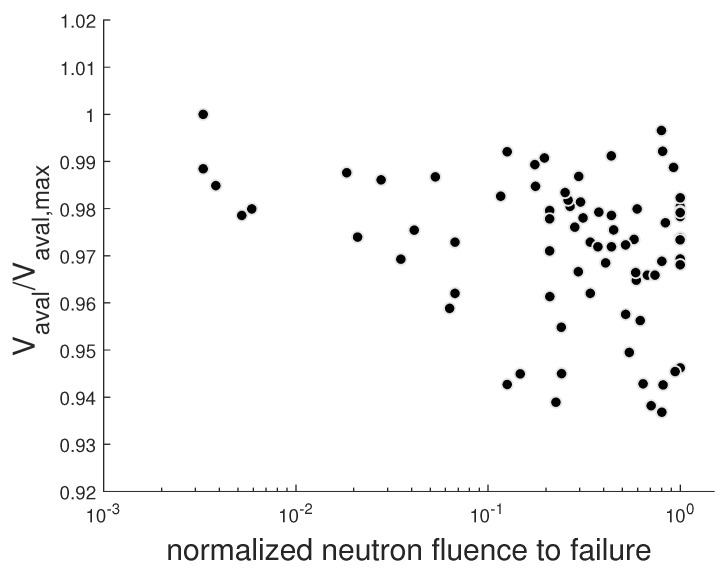
Scatter plot of the normalized $V_{aval}$ versus the normalized neutron fluence to failure of the SiC_A SiC MOSFET.

We now discuss the issue concerning the comparison of the FIT values of different devices as a function of the $V_{DS}$ voltage normalized to the avalanche voltage $V_{aval}$. In particular, we investigated the correlation between the $V_{aval}$ value and the probability of the failure event, to determine if SEB events under neutrons exposure were more likely in devices with lower $V_{aval}$ values. For a given irradiation run of the same part number device, we calculated the avalanche voltage values of each failed device normalized to the maximum value $V_{aval,max}$ calculated over all the failed devices. The neutron fluences to fail, in the same run, were normalized to their maximum value. To improve the statistics, we collected together the data of different runs of the same part number. In Figure 11, the result of this analysis for the SiC_A SiC MOSFET are shown. We note the lack of correlation between the avalanche voltage and the neutron fluence to failure for the SiC_A device. The same result was obtained for the other investigated devices. Therefore, the failure events were not more probable in devices with a lower $V_{aval}$ value.

#### 3.2.4. Effect of the Negative Gate Voltage

We performed irradiation runs of the SiC_A, SiC_D, and Si_E MOSFETs at some drain derating voltages with different negative $V_{GS}$ values. Neutron testing at the negative gate bias condition was stimulated by the observation that this operating condition is often used by SiC MOSFETs in several power applications. From the data of Table 4, the negative gate voltage did not increase the failure rate in different technologies and materials of MOSFETs with respect to the case of zero gate bias.

Therefore, the bias $V_{GS}=0$ V for cosmic ray tests could be considered the worst case condition for Si and SiC MOSFETs. In [8], commercial silicon power MOSFETs were observed to have no significant difference between burnout cross-sections measured at zero gate bias and those measured at full negative gate bias. The effect of the negative gate bias under neutron exposure was different than that occurring in heavy-ion and protons tests for silicon devices used in space applications, where the negative $V_{GS}$ increases the failure SEGR event with respect to the $V_{GS}=0$ V bias condition [29,30]. This could be explained by considering the different interaction mechanisms between charged particles and neutrons. Although neutrons and protons with the same energy induce the same charged fragments due to the spallation of silicon nuclei [4], protons in irradiation experiments are incident from an external source and not, as in case of neutron spallation, generated anywhere in the device volume with equal probability. Moreover, protons, unlike neutrons, will lose their kinetic energy via the electromagnetic interaction with the electron gas of the solid along the trajectory within the device.

## 4. Conclusions

Accelerated neutron tests were conducted to determine the robustness of SiC and Si power MOSFETs and IGBTs, fabricated with different technologies, against neutrons with energy ranging from thermal to ultra-fast. Up to 2000 devices were tested in the whole experiment to produce accurate statistical analysis.

Thermal neutrons, with a flux of $\approx 10^{12}$ times that of the terrestrial cosmic radiation at sea level, did not induce either SEB or degradations in the electrical performances of the devices even up to the rated drain and gate voltages. Therefore, despite the high thermal cross-section of boron-10, its low natural abundance (≈20% [8]) present in BPSG and the p-layers of Si devices were not enough to induce either degradation or SEB in the devices. Moreover, the LET of the ${}^{10}$B-neutron reaction products might not be enough to trigger SEB.

Fast neutrons (i.e., up to about 10 MeV) induce SEB failures in power devices only at the drain (collector) voltage close to the rated values, probably due to the activation of the $\left(n,p\right)$ and $\left(n,\alpha \right)$ nuclear reactions. For example, the SEB activation in the silicon power MOSFET Si_D occurred at $V_{DS}=560$ V and $V_{DS}=350$ V for fast and ultra-fast neutrons, respectively. In the cases of fast neutrons, the FIT values were several orders of magnitude lower than those obtained with ultra-fast neutrons.

The accelerated tests with ultra-fast neutrons (up to about 800 MeV) resulted in typical SEB failures of the devices. By comparing SiC and Si power MOSFETs with the same voltage rating, we observed that SiC devices exhibited significantly lower FIT than that of Si MOSFETs. Silicon devices showed a threshold drain voltage, below which no fails occurred. This threshold voltage was not observed in SiC MOSFETs. The FIT data of SiC MOSFETs, normalized both to the die size and avalanche voltage, although seeming to prove the existence of a common trend among some devices, were sensitive to the technology of the device. Moreover, in some cases, the curves FIT/cm${}^{2}$ versus $V_{DS}/V_{aval}$ showed differences that depended on the spread of the technological processes, which could obscure the impact of the device technology. The following results, regardless of neutron energy, were obtained:

- SEGR failure was not observed in all devices regardless of the gate and drain bias conditions.
- The power devices that survived the tests did not show degradation in the electrical parameters even when irradiated with gate bias close to the Fowler–Nordheim onset.
- For the same bias drain voltage, the negative gate bias did not increase the FIT values in MOSFETs with different technologies and materials.

## Figures and Tables

**Table 1:** List of the tested power devices.

Device	Part Number	Label	${\mathrm{BV}}_{\mathrm{DSS}}$ (V)
MOSFET SiC	GEN3 technology (under development)	SiC_A	1200
MOSFET SiC	SCT100N120G2D2AG	SiC_B	1200
MOSFET SiC	SCT30N120	SiC_C	1200
MOSFET SiC	SCT35N65G2V	SiC_D	650
MOSFET Si	STW12N120K5	Si_A	1200
MOSFET Si	STH2N120K5	Si_B	1200
MOSFET Si	STH22N95K5	Si_C	950
MOSFET Si	ST88N65M5	Si_D	650
MOSFET Si	STB45N40DM2AG	Si_E	400
IGBT	STG200M65F2D8AG	I_A	650
IGBT	STGW40H120DF2	I_B	1200
IGBT	STGW40H65DFB	I_C	650

**Table 2:** Neutron flux components at the end of the channel inside the thermal column of the TRIGA Mark II reactor upstream and downstream of the Boral shield.

Energy Range		Neutron Flux (cm${}^{-2}$ s${}^{-1}$)	
		Upstream of the Boral Shield	Downstream of the Boral Shield
Thermal	$E_{n}<$0.414 eV	1.6$\times 10^{9}\pm 0.2\%$	2.2$\times 10^{5}\pm 10\%$
Epithermal	0.414 eV$<E_{n}<$10.7 eV	6.2$\times 10^{6}\pm 3\%$	2.0$\times 10^{5}\pm 6\%$
Epithermal	10.7 eV$<E_{n}<$1.58 keV	6.3$\times 10^{6}\pm 3\%$	3.8$\times 10^{6}\pm 6\%$
Fast	1.58 keV$<E_{n}<$2 MeV	5.8$\times 10^{6}\pm 4\%$	5.2$\times 10^{6}\pm 5\%$
Fast	2 MeV$<E_{n}<$6 MeV	3.1$\times 10^{5}\pm 10\%$	2.7$\times 10^{5}\pm 10\%$
Fast	6 MeV$<E_{n}<$17.3 MeV	1.2$\times 10^{5}\pm 10\%$	1.2$\times 10^{5}\pm 10\%$

**Table 3:** Failure in time (FIT) at sea level of the MOSFET Si_D and IGBT I_B irradiated with fast neutrons.

	$V_{\mathrm{DS}}$ (V)	$V_{\mathrm{GS}}$ (V)	FIT	Lower 95% Confidence Limit	Upper 95% Confidence Limit
IGBT I_B	1100	0.0	0.33	0.09	0.72
MOSFET Si_D	560	0.0	0.56	0.35	0.82

**Table 4:** FIT values of some power MOSFETs measured in the zero and negative gate bias conditions.

MOSFET	$V_{\mathrm{DS}}$ (V)	$V_{\mathrm{GS}}$ (V)	FIT	Lower 95% Confidence Limit	Upper 95% Confidence Limit
SiC_A	950	0.0	1.06	0.55	1.73
	950	−4.0	0.62	0.27	1.12
SiC_D	480	0.0	4.25	2.48	6.50
	480	−4.0	4.28	2.45	6.63
Si_E	325	0.0	50.2	32.1	72.1
	325	−10.0	37.9	24.0	54.9
